# Abdominopelvic Actinomycosis Mimicking a Malignant Ovarian Neoplasia: Case Report and Review of Literature

**DOI:** 10.7759/cureus.12182

**Published:** 2020-12-20

**Authors:** Carlos A Regino, Kevin Navarro, Andrés García, Juliana Bacca, Natali Uribe

**Affiliations:** 1 Internal Medicine, University of Antioquia, Medellín, COL; 2 Internal Medicine, IPS Universitaria Clínica León XIII, Medellín, COL; 3 Pathology, University of Antioquia, Medellín, COL; 4 Infectious Disease, Pontifical Bolivarian University, Medellín, COL

**Keywords:** actinomycosis, actinomyces infections, ovarian neoplasm

## Abstract

Abdominal actinomycosis is a chronic, granulomatous, and indolent disease produced by *Actinomyces spp*., a gram-positive filamentous bacillus, anaerobic, commensal in the oral cavity, gastrointestinal tract, and pelvic mucosa. Diagnosis is usually difficult and delayed due to its insidious presentation. It can simulate different neoplastic, inflammatory as well as infectious diseases such as tuberculosis, nocardiosis, or mycosis. In most cases, the diagnosis is made postoperatively with the histopathological report, and only 10% of them are diagnosed preoperatively. We present two cases of abdominopelvic actinomycosis simulating advanced ovarian neoplasia.

## Introduction

Actinomycosis is an infrequent subacute to chronic infection caused by anaerobic or microaerophilic bacteria of the genus *Actinomyces spp* [1]. Due to current microbiological diagnostic technologies like mass spectrometry and 16s RNA genetic sequencing, 47 species have been identified of which 25 have caused disease in humans; the most commonly implicated is *A. israelii,* followed by *A. meyeri, A. naeslundii,* and *A. viscosus*. In 1970 the incidence rate in Cleveland in the United States was one per 300,000 person-years, compared to Germany and the Netherlands where it was estimated at one per million person-year [2]. In Latin America and Colombia, the evidence is limited to case reports [3]. However, between 2009 and 2013 around 400 cases have been reported per year [4]. Actinomycosis occurs throughout the world but is more common in countries with low income and poor health systems. It predominates in adults between 30 and 60 years, and is three times more common in men than in women, except for the pelvic form [5].

The *Actinomyces spp*. are members of the common human oral, gastrointestinal, and genital microbiota. Despite having a typically low growth rate and virulence, they can generate subsequent endogenous infection from mucosal disruption that occurs during trauma, intestinal necrosis, abdominal surgeries, endometrial curettage, and intrauterine device implantation (IUD) [6,7], as well as the presence of other foreign bodies such as pessaries and vaginal tampons [8]. Immunosuppressive states, such as diabetes mellitus, steroid therapy, and chronic inflammatory diseases favor disseminated disease [9].

In some cases, the inflammatory involvement can be so severe that it can mimic metastatic neoplastic disease. The objective of this article is to present two cases of abdominopelvic actinomycosis simulating advanced ovarian neoplasia and perform a literature review focused on the recognition, diagnosis and treatment of this disease.

## Case presentation

Case 1

**Figure 1:**
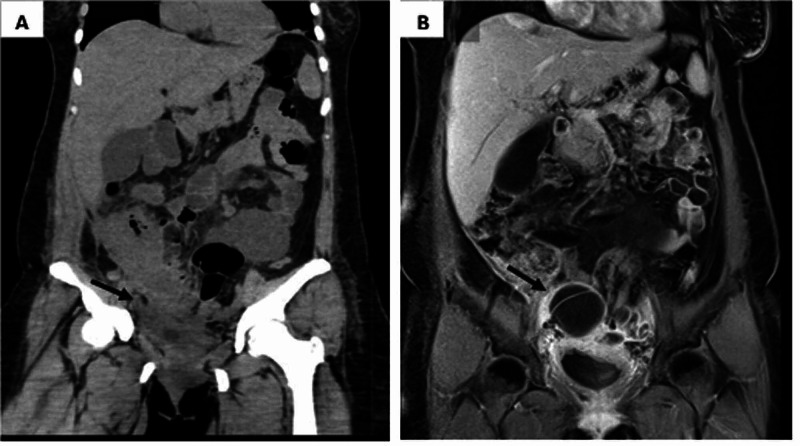
Abdominal tomography and magnetic resonance A: Abdominal tomography: A multicystic lesion that extends to the presacral region, associated with striation of pelvic fat, thickening of the bladder walls, and infiltration of the anterior abdominal wall. B: Abdominal magnetic resonance: Peritoneal inflammatory changes and right complex adnexal cyst.

**Figure 2:**
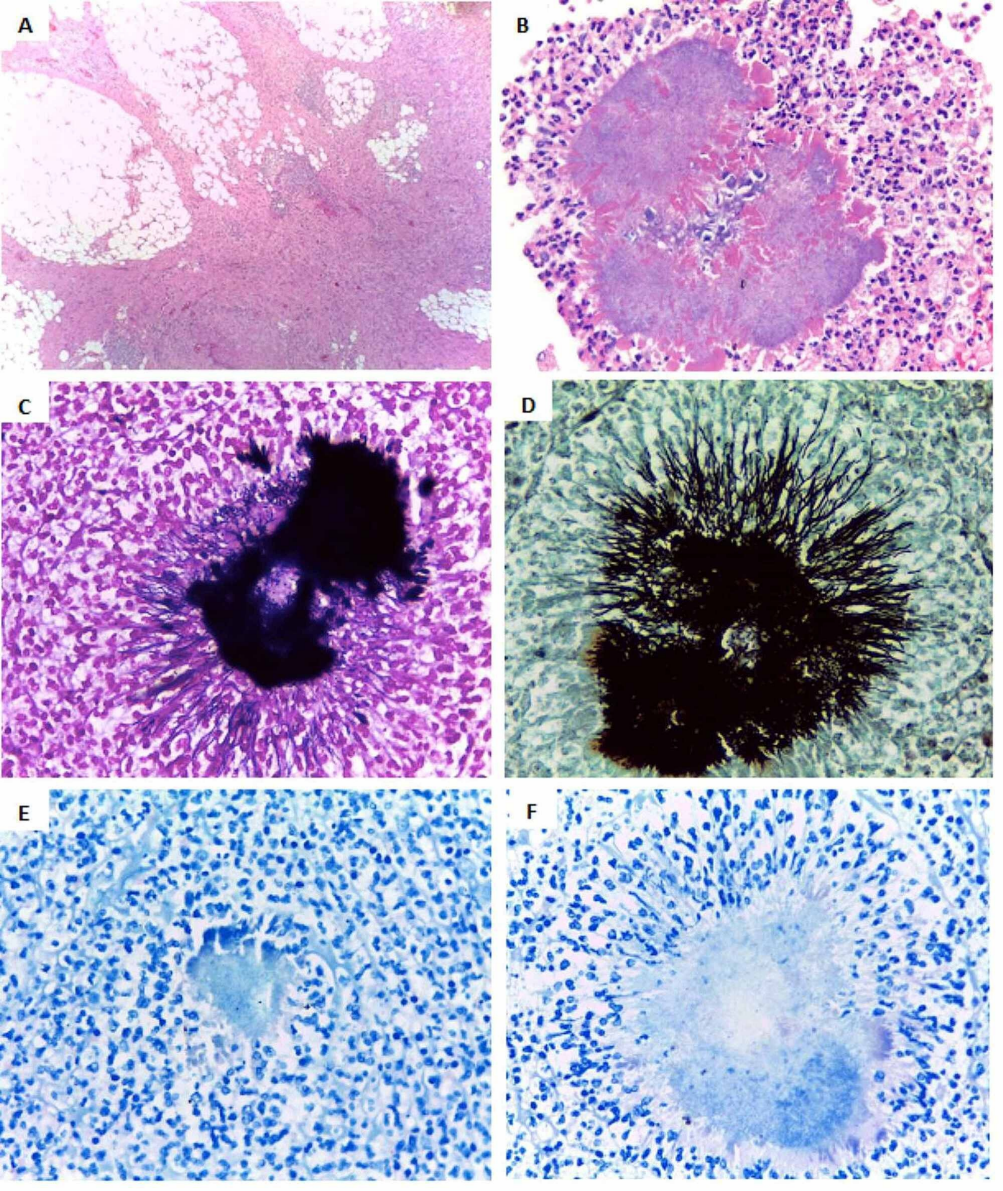
Pathology A) Mature fibroadipose tissue of the parietal peritoneum with chronic xanthogranulomatous inflammation. Hematoxylin-eosin staining 10x. B) *Actinomyces *spp. it is a large, radiated structure, made up of bacterial colonies of filamentous bacilli surrounded by an eosinophilic matrix called the Splendore-Hoeppli reaction, which corresponds to the protein exudate produced by bacteria. Hematoxylin-eosin staining 10x. C) Gram-positive bacilli. Gram staining 40x. D) Methenamine silver negative for fungi; in this case, positive silver filamentous bacilli are observed. E) Absence of acid-fast bacilli. Ziehl Neelsen stain 40x. F) Absence of atypical mycobacteria. Ziehl Neelsen staining modified 40x.

A 44-year-old woman with no medical history underwent an abdominal hysterectomy due to myomatosis. A year later, she consulted for 5 months of pelvic pain, vaginal discharge, intermittent fever, and weight loss of 30 kg. Physical examination revealed multiple abdominal masses. Tomographic studies showed collections in the peritoneal cavity with thick walls and enhancement with contrast (Figure 1a). MRI reported a complex, septate ovarian mass highly suggestive of advanced neoplastic disease (Figure 1b). Extension studies were negative for metastasis. A biopsy was performed under tomographic guidance showed foci of lymphoplasmacytic inflammatory infiltrate without the presence of granulomas or malignancy. Due to the high suspicion of malignancy, it was decided to perform open laparotomy with a new biopsy. Cultures for aerobes, anaerobes, fungi, and mycobacteria were negative, as well as the polymerase chain reaction (PCR) for M. tuberculosis (Gene/Xpert). The histopathological study showed abscessed chronic inflammation in the parietal peritoneum composed of a mixed inflammatory infiltrate with abundant neutrophils and foamy histiocytes accompanied by large filamentous radiated structures formed by gram-positive bacilli, negative for conventional and modified Ziehl-Neelsen, confirming the presence of *Actinomyces *spp. (figure 2). The postoperative course was uneventful. She received treatment with penicillin G 2.4 million IU every 24 hours for 10 days and then was discharged with oral amoxicillin for six months. To date, the patient shows no recurrence of the disease.

Case 2

**Figure 3:**
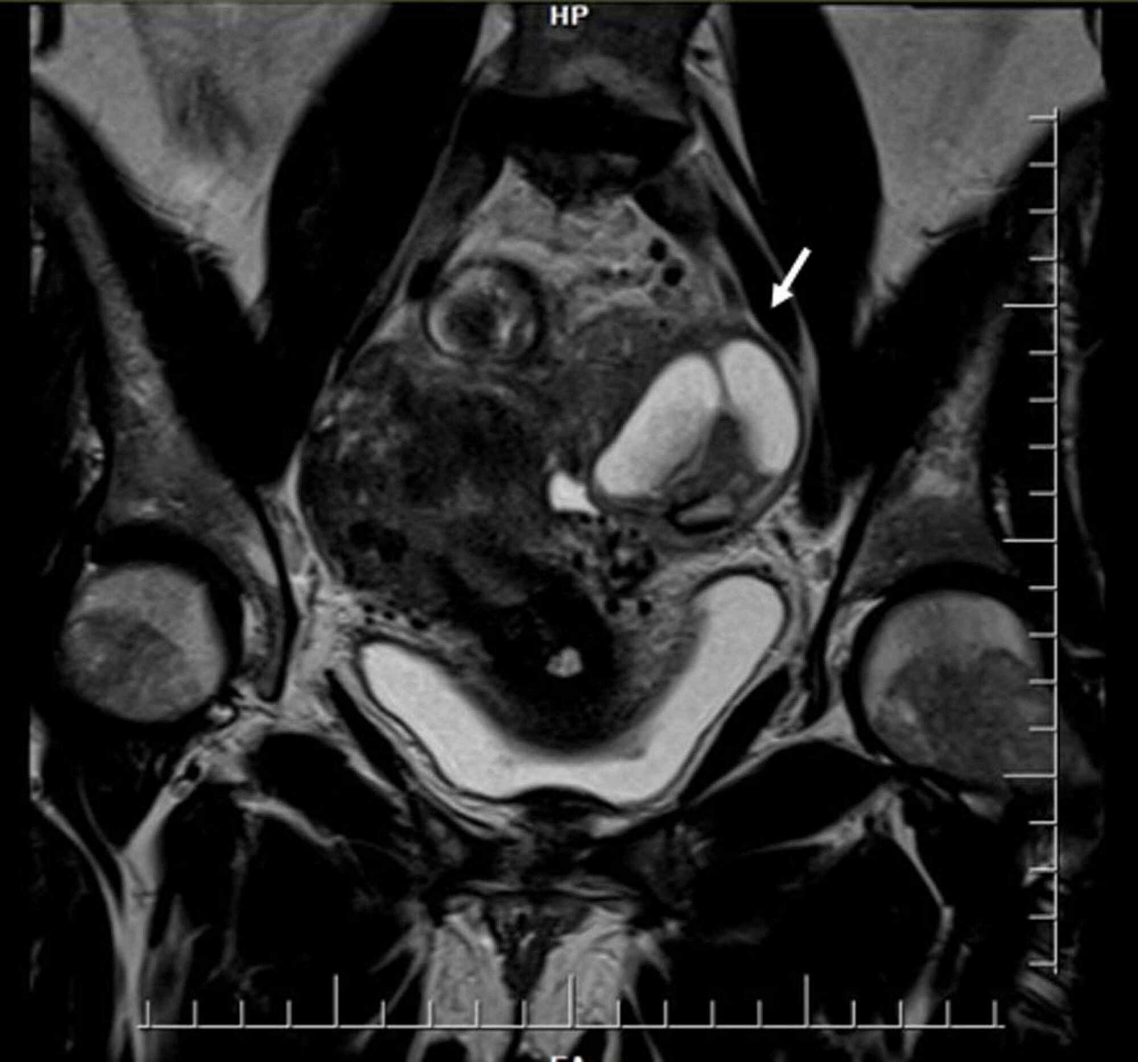
Abdominal magnetic resonance Complex left adnexal mass that contacts the left anterolateral wall of the upper rectum.

**Figure 4:**
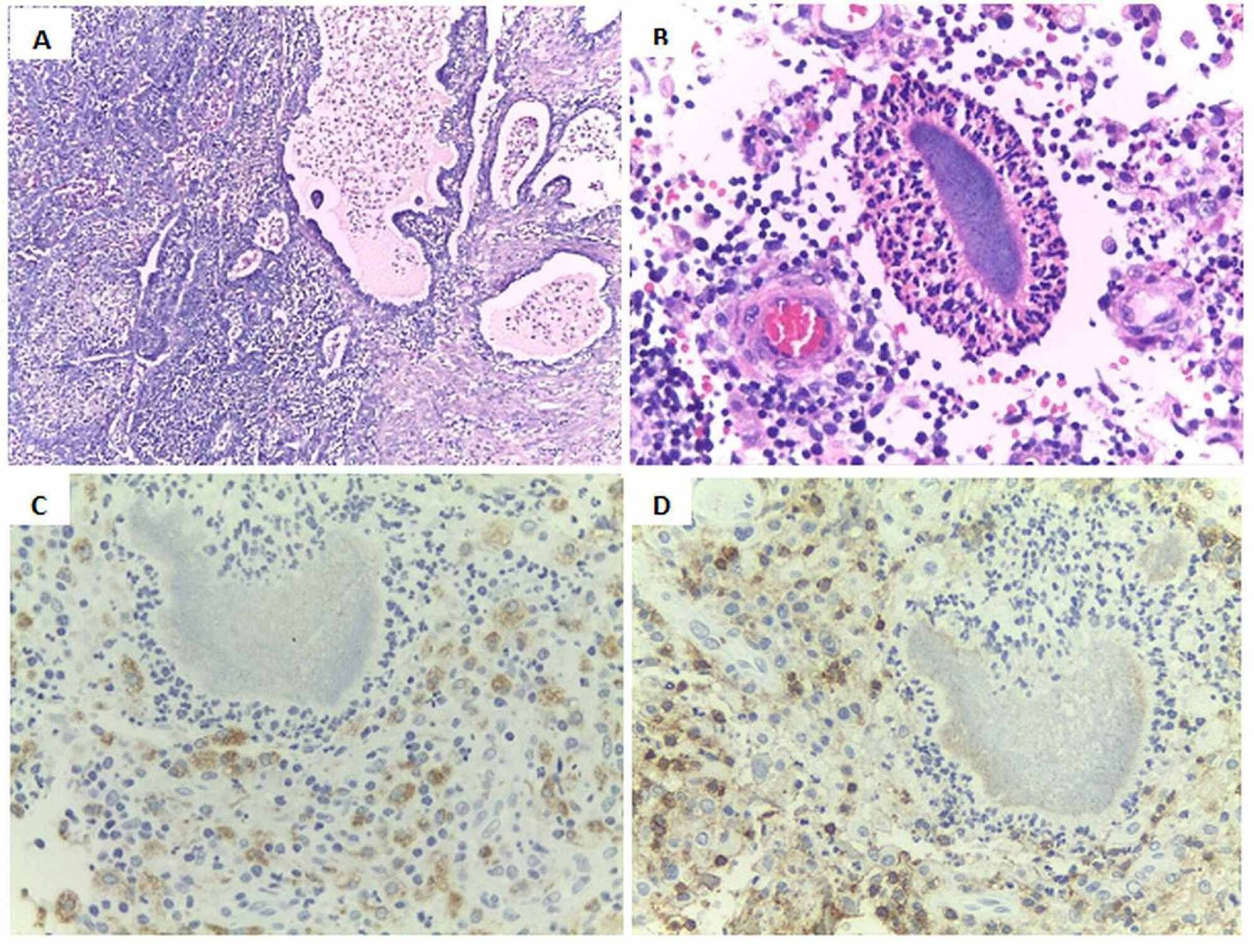
Pathology A) Uterine tube with non-dysplastic reactive epithelium, the stroma presents abundant foamy macrophages and a mixed inflammatory infiltrate of predominantly neutrophilic polymorphonuclear, constituting a chronic abscess inflammation. Hematoxylin-eosin staining 10x. B) *Actinomyces *spp*.* radiated, surrounded by the Splendore-Hoeppli reaction. C and D) Accompanying chronic inflammation typified by immunohistochemistry, showing CD68 pgm1 positive histiocytes (Figure C) and positive CD45 reactive B and T lymphocytes (Figure D).

A 46-year-old woman with no past medical history, with a history of cesarean delivery and intrauterine device contraception consulted for a one-year history of progressive pelvic pain and weight loss of 4 kg. Her clinical examination was unremarkable. An abdominal tomography was performed, which revealed a heterogeneous mass of 57×48×57 mm in the left adnexal, with a cystic component and solid areas associated with focal lesions in the intestinal wall that were interpreted as metastatic neoplastic disease. (figure 3). She underwent open laparotomy where multiple adhesions and an ovarian mass were found infiltrating the posterior aspect of the uterus with a significant inflammatory reaction. Intraoperative biopsy ruled out malignancy and the complete histopathological study reported alteration of the ovarian and tubal architecture due to extensive chronic inflammation with abscess formation where radiated cottony colonies were identified, surrounded by the Splendore-Hoeppli reaction compatible with *Actinomyces *spp (Figure 4). The postoperative period was uneventful; treatment was established with 2.4 million IU penicillin G every 24 hours for 14 days with a subsequent change to oral amoxicillin, which she received for six months. Her evolution was satisfactory with resolution of the disease.

## Discussion

Actinomycosis is an uncommon subacute or chronic progressive suppurative disease characterized by the formation of multiple abscesses, abundant granulation tissue, and dense fibrous tissue and may present mimicking findings consistent with either cervical or ovarian cancer [2]. Abdominal involvement classically manifests as a slow-growing mass in the ileocecal region in 65% of cases (less frequently in the stomach, duodenum, liver, rectum, or various organs), causing abdominal pain, weight loss, low-grade fever, nausea, and vomiting, making the preoperative diagnosis a clinical challenge [2,10].

Actinomycosis of the genital tract is observed more frequently in women who have used IUDs for more than 2 years [6,11]. Pelvic infection is characterized by nonspecific symptoms, the most common complaint being pelvic pain associated with vaginal bleeding or discharge [12]. The infection simulates different diseases such as neoplasms (and in the case of tubo-ovarian involvement, there is even an elevation of CA-125), tuberculosis, nocardiosis, and deep mycosis. In early stages, it presents as indolent infiltration, which leads to abscessation, fistulas, and fibrosis in the compromised organ; the presence of ascites, as well as the rapidly progressive evolution are exceptional [13]. Differential diagnoses include primary and metastatic neoplasms of the female genitourinary tract, endometriosis, and pelvic inflammatory disease caused by other microorganisms [10].

Diagnosis of actinomycosis is often difficult and delayed due to the insidious and prolonged course; only 4.5 to 10% of cases of abdominopelvic actinomycosis are diagnosed before surgery [9]. Laboratory findings show mild leukocytosis, increased levels of C-reactive protein, and erythrocyte sedimentation [14]. Computed tomography reveals a necrotic infiltrative mass, with or without the presence of lymphadenopathy, simulating a highly aggressive malignancy. Thus, the diagnosis is constructed with a correlation between the patient's signs and symptoms, anaerobic cultures, and histopathological findings [2,7]. In patients with high clinical suspicion and negative cultures, this disease is not ruled out since in 50% of cases, the cultures are negative. Therefore, the study of the biopsy or the specimen is necessary to confirm the presence of *Actinomyces *spp. and rule out other associated causes [15].

*Actinomyces *spp is made up of slow-growing, filamentous bacterial colonies that require prolonged anaerobic incubation of 5 to 20 days. Histologically, they are large, radiated structures surrounded by a proteinaceous reaction produced by the bacteria called Splendore-Hoeppli. This phenomenon is not specific to this entity since it can be observed in some bacterial and chronic parasitic infections, and in superficial and deep skin mycoses [13,15]. They are gram-positive bacteria, negative to conventional and modified Ziehl Nielsen stains, and methenamine silver lamination does not show fungal structures. Thus, other differential diagnoses such as *Nocardia *spp., *Mycobacterium leprae*, *Mycobacterium tuberculosis*, and deep mycosis are ruled out. One of the forms of presentation is actinomycotic granuloma, in which there are multinucleated giant cells and inflammatory cells, surrounded by a crown of histiocytes, in which there may be extensive areas of abscessation and fistulas. This inflammation produces reactive changes in the epithelia that should not be confused with dysplasia [8]. Finally, in rare cases, infection by *Actinomyces *spp. accompanies a malignant or benign neoplasm. Hence, proper processing of pathological samples is essential [16].

Treatment is based on high doses of antibiotics for a prolonged period. Penicillin G is the drug of choice in doses of 12-24 million international units per day for the cervicofacial presentation, 18-24 million for the thoracic and abdominopelvic form for 2 to 6 weeks, with subsequent oral therapy with penicillin V or amoxicillin for 6-12 months [13]. However, to define its adequate duration, imaging follow-up is generally used, thus reducing the possibility of relapses. Other regimens that have reported successful results include intravenous ampicillin followed by oral amoxicillin, or erythromycin, doxycycline, and clindamycin in case of allergy to penicillin [17]. In the second case, it should be taken into consideration that in some species, rates of in vitro resistance is up to 21.2% [18]. Several antibiotics have little or no activity against *Actinomyces* spp. among which we find metronidazole, aminoglycosides, aztreonam, cotrimoxazole, penicillinase-resistant penicillins (e.g., oxacillin), cephalexin, and fluoroquinolones [19]. Unpredictable resistance of certain *Actinomyces* strains to some antibiotics such as ceftriaxone, piperacillin/tazobactam, meropenem, and tetracyclines has been reported in the literature [20].

In general, concerning the prognosis of the disease, published studies have shown mortality that ranges between 0% and 28% mainly determined by the site of infection, the time until diagnosis, and the start of adequate treatment [2,10].

## Conclusions

Actinomycosis is a chronic disease difficult to diagnose due to its nonspecific clinical presentation, inflammatory compromise and its infiltrative nature that can simulate an advanced neoplastic lesion. It should be considered in the differential diagnosis of patients with recurrent abdominal pain and pelvic mass, particularly in women with a history of IUD use or abdominal or pelvic surgery. The diagnosis is usually made with histopathological findings and in less than half the cases, with the results of cultures. Treatment is based on high doses of parenteral antibiotics, followed by prolonged courses of oral antibiotics.
